# Suicidal ideation in patients with mental illness and concurrent substance use: analyses of national census data in Norway

**DOI:** 10.1186/s12888-021-03663-8

**Published:** 2022-01-04

**Authors:** Helle Wessel Andersson, Solfrid E. Lilleeng, Torleif Ruud, Solveig Osborg Ose

**Affiliations:** 1 Department of Research and Development, Clinic of Substance Use and Addiction Medicine, St. Olavs University Hospital, PB 3250 Sluppen, 7006 Trondheim, Norway; 2 Department of Analysis and Performance Assessment, The Norwegian Directorate of Health, Holtermanns vei 70, 7031 Trondheim, Norway; 3 Akershus University Hospital, Mental Health Services, PB 1000 1478 Lørenskog, Norway; 4 Institute of Clinical Medicine, University of Oslo, PB 1171 Blindern, 0318 Oslo, Norway; 5 Department of Health, SINTEF, Professor Brochs gate 2, 7030 Trondheim, Norway

**Keywords:** Suicidal ideation, Mental disorders, Substance use disorders, Substance use, Mental health services

## Abstract

**Background:**

Suicidal ideation may signal potential risk for future suicidal behaviors and death. We examined the prevalence of recent suicidal ideation in patients with mental illness and concurrent substance use and explored the clinical and sociodemographic factors associated with suicidal ideation in this patient subgroup, which represents a particular risk group for adverse psychiatric outcomes.

**Methods:**

We used national cross-sectional census data in Norway collected from 25,525 patients in specialized mental health services. The analytic sample comprised 3,842 patients with concurrent substance use, defined as having a co-morbid substance use disorder or who reported recent regular alcohol use/occasional illicit drug use. Data included suicidal ideation measured in relation to the current treatment episode, sociodemographic characteristics and ICD-10 diagnoses. Bivariate and multivariate analyses were used to examine differential characteristics between patients with and without suicidal ideation.

**Results:**

The prevalence of suicidal ideation was 25.8%. The suicidal ideation rates were particularly high for those with personality disorders, posttraumatic stress disorder, and depression, and for alcohol and sedatives compared with other substances. Patients with suicidal ideation were characterized by being younger, having single marital status, and having poorly perceived social relationships with family and friends.

**Conclusion:**

Suicidal ideation in patients with mental illness and concurrent substance use was associated with a number of distinct characteristics. These results might help contribute to an increased focus on a subgroup of individuals at particular risk for suicidality and support suicide prevention efforts in specialized mental health services.

## Background

Suicidal ideation (SI; thoughts of suicide) may signal a potential risk for future suicide attempts and completed suicide [1–3]. Therefore, as noted by several authors [4, 5] knowledge about the characteristics of individuals who have SI may contribute to the development of targeted interventions addressing this before it progresses to more severe acts.

Mental disorders are strongly associated with suicidal behaviors [6–8]. Clinical studies have repeatedly reported an increased risk of suicidality associated with mood disorders [9–13], personality disorders [10, 12, 13] and anxiety disorders [13, 14]. In patients with mental illness, concurrent alcohol or drug use may pose an elevated risk of suicidality [10, 15, 16].

Other risk factors for suicidality include being young and socially disadvantaged [8, 17]. However, research on the impact of sociodemographic factors on risk of suicidality among patients with mental illness has not always been conclusive. For example, among studies on risk factors for SI in the population undergoing mental health treatment, findings were inconsistent regarding educational level: one study found an association between SI and low education [18], while another did not [19]. Furthermore, two studies reported an association between SI and having poor social relationships [12, 19]; however, their results were divergent as to whether this relationship was independent of clinical factors.

Many of those who committed suicide had recently received treatment for mental disorders [3, 20–22]. Therefore, patients currently in specialized mental health treatment comprise an important target group for preventive suicidal measures. Individuals with concurrent substance use constitute a significant part of this patient population [15, 16, 23, 24]. Because individuals with mental illness are particularly vulnerable to negative consequences of substance use [24, 25], a further appropriate targeting of preventive measures would be to address SI in patients under psychiatric care with concurrent substance use.

While there are some studies on risk factors for SI among samples of psychiatric patients with diverse mental disorders [10–13, 16], we are aware of only two studies that examined the prevalence of SI in mentally ill patients with concurrent substance use. Both these studies were carried out among patients in acute psychiatric wards, and indicated an increased risk of suicidality associated with concurrent alcohol and drug use [10, 16]. As far as we know, no previous studies have investigated the clinical and sociodemographic factors associated with SI in general psychiatric patients with concurrent substance use.

Characterizing those who have SI among patients with mental illness and concurrent substance use may be helpful in identifying individuals at suicide risk and contribute to developing suicide prevention efforts within mental health services. Accordingly, the aim of this study was to examine the prevalence of recent SI in psychiatric patients with concurrent substance use by using cross-sectional national census data on unselected patients in specialized mental health services in Norway. We have identified and characterized this subsample in a previous publication. Our earlier study showed that compared with patients without substance use, those with concurrent substance use were more socially marginalized (Andersson et al., 2020). This may pose an additional risk for suicidality in this already vulnerable patient group. A particular aim of the current study was to examine the prevalence of and types of mental disorders and substance use associated with recent SI, and the association between sociodemographic factors and SI in this subgroup of patients under psychiatric care.

## Methods

### Setting and design

The data for our study originate from two datasets based on two national cross-sectional censuses of patients being treated by specialized psychiatric services in Norway. The study setting and design have been described previously [26]. Briefly, one of the censuses was conducted in inpatient wards and departments (including acute wards) to include all patients available on a specific date (November 20, 2012), while the other included all patients receiving treatment at outpatient clinics during a 14-day period (April 15–28, 2013). The two censuses used the same procedures, which justified combining the two datasets to create a gross sample for the current study.

The patients’ clinicians were responsible for filling in an anonymous questionnaire for each case based on information from their medical records supplemented with information provided by the patient as part of the study. The questionnaire included data on sociodemographic characteristics, mental health and substance use disorders (SUD) concurrent substance use, social relationships, and suicidality. The Regional Committee for Medical and Health Research Ethics approved the study (reg. no. 2012/848).

### Variables

#### Sociodemographic characteristics

The sociodemographic variables included were age, gender, educational attainment, income, marital status, housing situation and social relationships. Educational attainment was graded as low level (only primary school), medium level (secondary school), or higher level (university or higher education). Income encompassed income from labor, health-related benefits and other economic support. Marital status was classified as: married/cohabitant/partner, separated/divorced/widow(er), or single/unmarried. Housing situation was classified as: living in owned home, living in rented housing, or without permanent residence (incl. living with family or friends). Information on social relationships was based on the patients’ responses to the following two questions: “How is your relationship with your family?” and “How is your relationship with friends?” The questions were answered on a 4-point scale (“very good,” “quite good,” “quite bad,” and “very bad.”

#### Mental disorders

Information on the main psychiatric diagnosis according to the International Classification of Diseases, ICD-10 [27] was collected from the patients’ medical records. The diagnoses were grouped into the following binary variables (1 = presence 0 = absence): schizophrenia (F20), other psychoses (F22–F25, F28–F29), bipolar disorders (F31), depression (F32–F33), anxiety (F40, F41), eating disorders (F50), personality disorders (F60, F61), and other psychiatric diagnoses or unspecified (i.e., all other F-diagnoses).

#### Concurrent substance use

Based on the medical records, the clinicians reported information on the type of SUD diagnosis (ICD-10, F10–F19) recorded as an additional diagnosis (up to two additional diagnoses could be coded). In addition, the clinicians collected data on the patients’ substance use (alcohol and/or drugs) during the four weeks preceding treatment (up to three different types of substances used could be recorded), including frequency of use. We defined patients with concurrent substance use as those having either a SUD diagnosis or who reported regular alcohol use/occasional illicit drug use in the last four weeks. Type of concurrent substance used was categorized into the following six binary variables (1 = presence; 0 = absence): alcohol (F10), opioids (F11), cannabis (F12), sedatives (F13), stimulants (F14, F15), or multiple substance use (F19). These criteria for concurrent substance use and characteristics of the sample have been described previously (Andersson et al., 2020).

#### Suicidal ideation

Suicidal ideation was identified through a question developed for the National census. The clinicians who were responsible for filling out the mapping form for their patients’ were asked to report whether the patient had had any kind of suicidal behavior in the last four weeks. The response options were: “no suicide risk behaviors,” “suicidal thoughts,” “suicide threats,” and “suicide attempts.” For the current study, we defined patients with recent SI as those having either suicidal thoughts, threats, or attempts (1 = yes), and no SI as those without any suicidal thoughts or behaviors (0 = no). Measuring SI by combining thoughts, threats, or attempts is justified by research suggesting that the risk profile of individuals with suicidal thoughts is the same as for those with suicidal behaviors [28, 29].

### Statistical analyses

First, based on the gross sample, we compared rates of SI between patients with and without concurrent substance use using chi-square tests. Further analyses were restricted to patients with concurrent substance use (i.e., the analytic sample). Chi-square tests were employed to assess the difference in distribution of the categorical variables (sociodemographic variables and psychiatric diagnoses) between patients with and without SI. Because the psychiatric treatment level (inpatient versus outpatient) might reflect the severity of patients’ mental health conditions and sociodemographic characteristics [23] that can influence the prevalence of SI [11], this variable was included in the analysis. We used binary logistic regression analysis to compare the proportion of SI in each substance use category with the proportion of SI in all other substance use categories combined. The estimated odds ratio (OR) and 95% confidence intervals (CI) with a p-value < 0.05 indicated that rate of SI in the given substance use category was significantly different from the SI rate in all other substance use categories combined.

To explore the clinical and sociodemographic factors that contributed the most to the difference between patients with and without SI, a multivariate logistic regression was constructed, including variables associated with SI at p < 0.05 in bivariate analysis. All analyses were performed using STATA (Stata/SE 16 for Windows; StataCorp LP, College Station, TX, USA).

## Results

### Sample characteristics

The gross sample comprised 25,525 patients (23,167 outpatients and 2,258 inpatients,) estimated to cover 60% to 65% of patients in Norwegian specialized psychiatric services at that time (see Ose et al., 2017). Data on suicidality were missing for 1,265 patients (5%) and these cases were excluded from the study. The analytic sample consisted of 3,842 patients (751 inpatients and 3,091 outpatients) with concurrent substance use (of whom 30% with a SUD diagnosis). The analytic sample was the same as that identified and described earlier [23], except that individuals with missing data on suicidality were excluded from the current study (*n* = 131). Briefly, the sample comprised 56% men and 44% women, and most were aged 30–39 years (23%) or > 50 years (23%). Most had a low educational level (44%) and a source of income other than from employment (83%). In total, 992 (25.8%) of the patients had SI (suicidal thoughts: 80%; threats: 12%; attempts: 8%). The SI rate of patients with concurrent substance use was significantly higher than that of nonusers (25.8% vs 18.3%; p < 0.001; Table 1).

**Table 1:** Prevalence of suicidal ideation in gross sample and according to patients’ substance use status

Suicidal ideation status	Gross sample (*n* = 25 525)		Substance use^a^ (*n* = 3842)		No substance use (*n* = 20 413)		Substance use vs no substance use		
	n	%	n	%	n	%	χ^2^	df	*p*-value
Suicidal ideation	4 730	19.5	992	25.8	3 738	18.3			
No suicidal ideation	19 530	80.5	2850	74.2	16 680	81.7			
Chi-square test							109.7	1	< 0.001

Total number with missing data on suicidality: *n* = 1 265, ^a^ Analytic sample: inpatients, *n* = 751; outpatients *n* = 3091

### Bivariate analyses of factors associated with recent SI

Table 2 presents the clinical and sociodemographic characteristics of patients with and without SI, and the chi-square results for comparisons between these groups. The results of the bivariate analyses revealed that rates for SI varied considerably between the eight specified diagnostic groups. It was most prevalent in patients diagnosed with personality disorders (45.1%), posttraumatic stress disorder (PTSD; 39.3%), and depression (37.7%). Further, some but not all sociodemographic categories were significantly associated with the presence of SI. The SI rate was higher in female (27.2%) than in male subjects (24.0%), and higher in patients aged 18–23 years (33.7%) compared with older age groups. The presence of SI was also associated with marital status and was more common among separated/divorced patients (30.0%) than among those who were married (23.2%). Furthermore, SI occurred more frequently in patients who perceived having very poor social relationships to their family (34.5%) and friends (30.4%) compared with those who perceived good or very good social relationships. The remaining sociodemographic variables included in the analyses—education level and main source of income—were not associated with the presence of SI. The treatment-level variable included in the analyses showed that the SI rate was considerably higher among inpatients (33.3%) than outpatients (24.0%).

**Table 2:** Rates of suicidal ideation according to clinical and sociodemographic characteristics

Variables			Suicidal ideation rates	Chi-square test		
	N	%	%	χ^2^	df	*p*-value
***Diagnoses***				175.14	8	< .001
Schizophrenia	654	17.0	14.4			
Other psychoses	224	5.8	15.6			
Bipolar disorder	249	6.5	23.3			
Depression	624	16.2	37.7			
Anxiety	288	7.5	19.1			
PTSD	145	3.8	39.3			
Eating disorders	68	1.8	19.1			
Personality disorders	264	6.9	45.1			
Other psychiatric disorders	1326	34.5	24.6			
***Gender***				5.02	1	.025
Male	2033	55.6	24.0			
Female	1621	44.4	27.2			
***Age group***				30.34	4	< .001
18–23 years	572	15.3	33.7			
24–29 years	712	19.1	26.5			
30–39 years	872	23.4	22.6			
40–49 years	729	19.5	28.3			
> 50 years	846	22.7	22.3			
***Education***				3.21	2	.201
High	564	15.6	29.1			
Medium	1481	40.9	25.7			
Low	1579	43.6	25.3			
***Maine source of income***				3.14	2	.208
From labor	652	17.3	27.3			
Health related benefit	2496	66.4	24.8			
Other economic support	613	16.3	27.7			
***Marital status***				8.49	2	.014
Married/cohabitating/partnered	948	24.9	23.2			
Separated/divorced/widowed	570	15.0	30.0			
Single/unmarried	2288	60.1	25.7			
***Housing situation***				1.76	2	.415
Owned home	1317	34.9	24.7			
Rented housing	1665	44.1	26.7			
Without permanent residence	791	21.0	25.2			
***Social relationships family***				69.75	3	< .001
Very good	963	26.4	17.4			
Good	1657	45.4	25.5			
Poor	680	18.6	33.2			
Very poor	351	9.6	34.5			
***Social relationships friends***				20.33	3	< .001
Very good	644	18.0	20.5			
Good	1578	44.0	25.1			
Poor	845	23.6	29.0			
Very poor	519	14.5	30.4			
**Treatment level**				26.1	1	< .001
Inpatients	751	19.5	33.3			
Outpatients	3091	80.5	24.0			

As shown in Table 3, the rates of SI also varied by types of substance use. The SI rates were significantly higher for patients regularly using alcohol (OR = 1.17; p < 0.05) and for those using sedatives (OR = 1.31; p < 0.001) compared with users of other substances. Patients who were using cannabis were less likely to experience SI (OR = 0.84; p < 0.05) compared with users of other substances, as were those using stimulants (OR = 0.69; p < 0.001). Many of the patients had more than one SUD diagnosis or more than one substance recorded, indicating combination substance use. A closer look at the data showed that the two most common combinations of substances used was cannabis and stimulants (309 cases) followed by alcohol and cannabis (200 cases).

**Table 3:** Rates of suicidal ideation according types of substances used^a^

Types of substances	Patients	%^a^	OR^b^	95% CI		*p*-value
Alcohol (F10)	1645	27.5	1.17	1.011	1.352	0.036
Opioids (F11)	200	25.0	0.96	0.688	1.327	0.785
Cannabis (F12)	1169	23.6	0.84	0.720	0.991	.037
Sedatives (F13)	1071	29.7	1.31	1.122	1.538	< 0.001
Stimulants (F15)	727	20.5	0.69	0.571	0.846	< 0.001
Multiple substance use (F19)	1049	24.4	0.90	0.766	1.063	0.217

^a^Proportion of patients with suicidal ideation within each substance use category

^b^The likelihood of SI in the given substance use category (1) compared with the likelihood in all other substance use categories combined (0)

### Results of multivariate analyses

Table 4 provides the OR values calculated from the multivariable logistic regression model for variables associated with SI in bivariate analyses (Tables 2 and 3). Compared with individuals with a diagnosis of schizophrenia, all of the specified diagnoses, except other psychoses and eating disorders, were associated with higher odds of SI. The odds of SI was particularly high for patients with a personality disorder (OR = 4.59), with PTSD (OR = 4.64) 4.49), or with depression (OR = 4.45).

**Table 4:** Multivariate analyses of factors associated with suicidal ideation

Variables	OR	95% CI		*p*-value
Diagnoses: Base: Schizophrenia				
Other psychoses	1.19	0.742	1.906	0.472
Bipolar disorder	2.15	1.391	3.323	0.001
Depression	4.45	3.157	6.260	< .001
Anxiety	1.98	1.303	3.016	0.001
PTSD	4.64	2.923	7.377	< .001
Eating disorders	1.64	0.800	3.350	0.177
Personality disorders	4.59	3.107	6.784	< .001
Other psychiatric disorders	2.15	1.589	2.909	< .001
Types of substances used: Dummies^a^				
Alcohol (F10)	1.35	1.094	1.676	0.005
Cannabis (F12)	0.90	0.720	1.137	0.389
Sedatives (F13)	1.50	1.203	1.871	< .001
Stimulants (F15)	0.75	0.588	0.963	0.024
Gender: Base: Male				
Female	1.01	0.842	1.207	0.930
Age: Base: > 50 years				
18–23 years	2.73	1.964	3.803	< .001
24–29 years	2.17	1.602	2.949	< .001
30–39 years	1.60	1.195	2.131	0.002
40–49 years	1.81	1.376	2.371	< .001
Marital status: Base: Married/ partner				
Separated/divorced/widow(er)	1.75	1.322	2.310	< .001
Single/unmarried	1.24	0.993	1.547	0.058
Family relationships: Base: Very good				
Good	1.44	1.147	1.821	0.002
Poor	2.09	1.590	2.735	< .001
Very poor	2.22	1.590	3.105	< .001
Friends relationships: Base: Very good				
Good	1.18	0.912	1.522	0.209
Poor	1.31	0.975	1.748	0.073
Very poor	1.54	1.106	2.158	0.011
Treatment level: Base: Outpatient				
Inpatient	2.02	1.607	2.550	< .001
Constant	0.03	0.016	0.048	< .001

*Log likelihood* = *-1706.9628, Chi* = 308.34 (*p* < .0001), *N* = 3274

^a^The proportion of SI in the given substance use category (1) compared with the proportion of SI in all other substance use categories combined (0)

The substances associated with SI in bivariate analysis remained significant in the multivariate model. Compared with all illicit substances, alcohol was associated with a higher odds of SI (OR = 1.35). Compared with alcohol and other illicit drugs, sedatives were associated with a higher odds of SI (OR = 1.50). Stimulant use was associated with a lower odds of SI (OR = 0.75), compared with all other substances.

Among the sociodemographic variables included in the model, age, marital status, and social relationships with friends and family remained significantly associated with having SI. The odds of experiencing SI decreased with age and was highest for individuals aged 18–23 years (OR = 2.73). Compared with patients who were married, being separated was associated with higher odds of SI (OR = 1.75). Compared with patients reporting very good family relationships, the odds of SI increased slightly for each of the alternative response categories: good (OR = 1.44), poor (OR = 2.09) or very poor (OR = 2.22). Furthermore, SI occurred more frequently in patients who perceived having very poor friends relationships (OR = 1.54) compared with those reporting very good friend relationships.

## Discussion

This study adds to existing research examining clinical and sociodemographic factors associated with SI in psychiatric patients with concurrent substance use. We identified several clinical and sociodemographic characteristics associated with SI in this significant subgroup of patients undergoing mental health treatment.

We found that one in four psychiatric patients with concurrent substance use had SI, and the SI rate was considerably higher than that among patients with no substance use. We are not aware of any directly comparable studies; however, our data are consistent with previous research conducted on unselected acutely mentally ill patients that suggested an elevated risk of suicidality in patients with current alcohol or drug use [10, 15, 16]. The higher SI rate found among inpatients compared with outpatients was in accord with a previous finding [11], and could be related to different patient compositions found in outpatient versus inpatient units, including more adverse sociodemographic characteristics among inpatients [23].

The most directly comparable research to the present one regarding SI risk associated with diverse mental disorders have been studies conducted among unselected psychiatric samples using similar methodologies with the current study in measuring SI. Our findings of particularly elevated rates of SI associated with depression and personality disorders agree with those previous studies [10, 12, 13]. Moreover, this finding is unsurprising given the higher risk of suicide associated with SUD in these diagnostic groups [30, 31]. In addition, our results showed an increased risk of SI among patients diagnosed with PTSD. Previous clinical research has presented mixed results regarding the association between anxiety disorders and SI, possibly because the studies varied in how they defined the diagnostic groups [11–14]. A comprehensive literature review and meta-analysis that included both clinical and general samples suggested that among the anxiety disorders, PTSD was significantly associated with both SI and attempts at suicide [14]. Thus, the risk of suicidality associated with PTSD might have been undetected in previous mental health treatment studies that analyzed the broader ICD-10 category of anxiety disorders [11–13]. Previous research suggested that patients with both SUD and PTSD exhibit more severe substance use [32] and adverse treatment outcomes [33]. Our findings suggest that PTSD and related emotion dysregulation in combination with substance abuse might be associated with an elevated psychological burden and an increased risk of SI in patients under psychiatric care.

Our results showed that alcohol and/or sedatives use were associated with an increased risk of SI compared with other types of drugs (e.g., opioids, cannabis, or stimulants), irrespective of the type of mental illness and sociodemographic characteristics. Both alcohol and sedatives (e.g., benzodiazepines) are central nervous system depressants associated with an elevated risk of suicidal behaviors, compared with other types of drugs [34]. In patients with mental disorders, especially depression, use of alcohol may indicate greater serotonergic impairment, which might be associated with more negative mood states and increased risk for suicidality [35]. Moreover, in this population, the use of benzodiazepines in particular might be associated with an increased risk of suicidality [36]. On the other hand, among mentally ill patients with SI, readily available substances such as alcohol and sedatives may be used as a means of reducing psychological pain and psychosocial stressors associated with the mental disorder and a difficult life situation. Adverse psychosocial factors remained independently associated with SI in the multivariate model, which supports this latter assumption. The current findings of an increased SI risk associated with adverse sociodemographic factors align with previous findings from mental health treatment samples [10, 12, 13, 18, 19]. Our study contributes to existing knowledge by suggesting that among patients with concurrent substance, those with younger age, single marital status and poor social relationships may have a further elevated SI risk. Based on the current findings, it may be suggested that measures aimed at those who suffer social marginalization may reduce suicidal thoughts and future suicide attempts in this particularly vulnerable patient group.

### Strengths and limitations

A main strength of the current study was the large sample size, including both outpatients and inpatients with concurrent substance use in specialized mental health care clinics. The study focused on a significant subgroup of patients undergoing specialized mental health treatment, who, despite their increased risk of severe psychiatric outcome [24, 25, 37], have received relatively little attention in previous research on SI. The large sample size enabled analyses with a relatively detailed categorization of patients according to types of mental disorders and substances used. The study also included relatively detailed information on sociodemographic characteristics, some of which have been considered by only a few previous studies of SI among patients with mental disorders.

The study had several limitations. We combined datasets from cross-sectional censuses conducted in psychiatric inpatient and outpatient wards and departments. Because of a time gap of about 5 months between the two censuses, a patient who were included in the mapping while in inpatient treatment may later have received outpatient treatment. According to a Norwegian report from the census, 8% of the outpatients received inpatient treatment during the last quarter of the previous year [38]. Hence, a maximum of 247 outpatients in the current study could potentially been mapped twice. This means we cannot rule out a potential bias of dependent observations. The cross-sectional design of the study precluded any causal inferences. The study included several non-validated measures. Information on SI in the patients was based on the clinicians’ reports, as has been done previously [16]. According to national guidelines for prevention of suicide in specialized mental health services, the clinicians should routinely perform suicide risk assessment as part of the psychiatric evaluation [39]. Because the use of such routinely collected data is appropriate, especially in conducting large-scale studies, future research on suicidality among patients under psychiatric care should examine the validity of the medical record information on suicidality provided by clinicians. Further, concurrent substance use was defined as having a SUD diagnosis or recent regular alcohol use/occasional drug use. While a SUD diagnosis was based on ICD-10 criteria, information on alcohol/drug use was based on the patient-reported substance use data collected by the responsible clinician. The use of standardized screening instruments for substance use, such as for example the alcohol and drug use identification tests (AUDIT and DUDIT), might have provided more valid measures of substance use in this study. The current study also used a non-validated scale to assess the patients’ ratings of their relationships with friends and family. Because social relationships appear to play an important role in the prevalence of SI in this population, future research could examine the significance of this finding in more detail using validated instruments.

## Conclusion

Patients with mental illness and concurrent substance use have an increased probability of experiencing SI. The risk of SI is particularly high in patients diagnosed with personality disorder, PTSD or depression. In addition, our findings suggest that patients who are socially marginalized, or below 30 years of age may have a further elevated SI risk. Patients with these characteristics might need special attention to prevent future more severe forms of suicidal behaviors.

## Data Availability

The data that support the findings of this study are available from author SOO upon reasonable request.

## Abbreviations

SI: Suicidal ideation
SUD: Substance use disorders
PTSD: Posttraumatic stress disorder
ICD-10: International Statistical Classification of Diseases and Related Health Problems 10^th^ version
WHO: World Health Organization
OR: Odds ratio
CI: Confidence interval
AUDIT: Alcohol Use Disorders Identification Test
DUDIT: Drug Use Disorder Identification Test
